# Daily forecast of dengue fever incidents for urban villages in a city

**DOI:** 10.1186/1476-072X-14-9

**Published:** 2015-01-31

**Authors:** Ta-Chien Chan, Tsuey-Hwa Hu, Jing-Shiang Hwang

**Affiliations:** Research Center for Humanities and Social Sciences, Academia Sinica, 128 Academia Road, Section 2, 115 Nankang, Taipei, Taiwan; Institute of Statistical Science, Academia Sinica, 128 Academia Road, Section 2, 115 Nankang, Taipei, Taiwan

**Keywords:** Logistic regression, Dengue, Dynamic threshold, Decision support

## Abstract

**Background:**

Instead of traditional statistical models for large spatial areas and weekly or monthly temporal units, what public health workers urgently need is a timely risk prediction method for small areas. This risk prediction would provide information for early warning, target surveillance and intervention.

**Methods:**

Daily dengue cases in the 457 urban villages of Kaohsiung City, Taiwan from 2009 to 2012 were used for model development and evaluation. There were in total 2,997 confirmed dengue cases during this period. A logistic regression model was fitted to the daily incidents occurring in the villages for the past 30 days. The fitted model was then used to predict the incidence probabilities of dengue outbreak for the villages the next day. A percentile of the 457*30 fitted incidence probabilities was chosen to determine a cut-point for issuing the alerts. The covariates included three different levels of spatial effect, and with four lag time periods. The population density and the meteorological conditions were also included for the prediction.

**Results:**

The performance of the prediction models was evaluated on 122 consecutive days from September 1 to December 31, 2012. With the 80th percentile threshold, the median sensitivity was 83% and the median false positive rate was 23%. We found that most of the coefficients of the predictors of having cases at the same village in the previous 14 days were positive and significant for the 122 daily updated models. The estimated coefficients of population density were significant during the peak of the epidemic in 2012.

**Conclusions:**

The proposed method can provide near real-time dengue risk prediction for a small area. This can serve as a useful decision making tool for front-line public health workers to control dengue epidemics. The precision of the spatial and temporal units can be easily adjusted to different settings for different cities.

## Background

The increasing economic and disease burden of dengue not only is an important public health issue in tropical and sub-tropical countries [1], but it has also become an increasing threat to intemperate countries in Europe and North America due to the effect of rising temperatures [2, 3]. The World Health Organization (WHO) estimated that there are 50–100 million dengue infections globally every year, including 500,000 people with severe conditions that require hospitalization, and about 2.5 % of those severe cases die [4]. One study in Southeast Asia found that the annual economic burden was US$950 million (95% certainty level: US$610 million- US$1,384 million) and disability-adjusted life years (DALYs) per million inhabitants was 372 (210–520) [5]. Dengue is a vector-borne viral disease transmitted by vectors such as *Aedes aegypti* and *Aedes albopictus* to humans. So far, environmental cleaning or peridomestic space spraying of insecticides have been the two major approaches for controlling and preventing dengue epidemics in communities [6]. Although insecticide resistance is a critical problem for controlling epidemics or outbreaks [7], the combined strategy of proper chemical spraying [8] and removal of vector-breeding sites [9] can indeed minimize risks of dengue infection. In addition to such control strategies, public health workers urgently need to know where the next high-risk areas are in order for them to monitor intensively, identify the cases early, and clean out the environment as soon as possible.

Therefore, the dengue surveillance involves routine collection on both the clinical infection cases and mosquito density, such as the House Index (HI), Container Index (CI) and Breteau Index (BI) [10]. However, because the mosquito surveillance is not systematic and does not collect data in real time, this indicator is not very sensitive or reliable for risk prediction [11]. In the empirical findings, environmental factors such as temperature and precipitation are correlated with the dengue virus’ activities and vector’s life cycle [12–14]. The other challenge for dengue prevention is the unpredictability of human movements. Unlike people, mosquitoes tend to have short movement distances (i.e. flying mostly shorter than 150 meters) [15]. Moreover, the infected persons usually find it difficult to recall where they got their mosquito bites. Therefore, we can only use the location of confirmed dengue cases coupled with the incubation time lag to differentiate the possible previous local or neighboring effect on the future risk of a dengue outbreak. In addition, the underlying transportation network between the sites of infection (i.e. where the person was bitten) and their residence might be estimated from the cumulative spatio-temporal trajectory of the confirmed cases. House-to-house human interaction is an important factor in dengue virus transmission [16]. Population density may also play a role in increasing the risk of dengue infection [17]. It would be beneficial to consider all these factors to enhance the accuracy of risk prediction.

Instead of traditional statistical models for large spatial areas and weekly or monthly temporal units, what public health workers need is a timely and local risk prediction method. The alert threshold should evolve with time, and the risk level should be adjusted throughout the different stages of an epidemic. This method should balance accuracy and false positives, and can help with early warning, the allocation of resources, risk communication and disease control. This study proposes a simple and timely method to predict incidence probability of dengue outbreak at a local level.

## Methods

### Data

**Figure 1:**
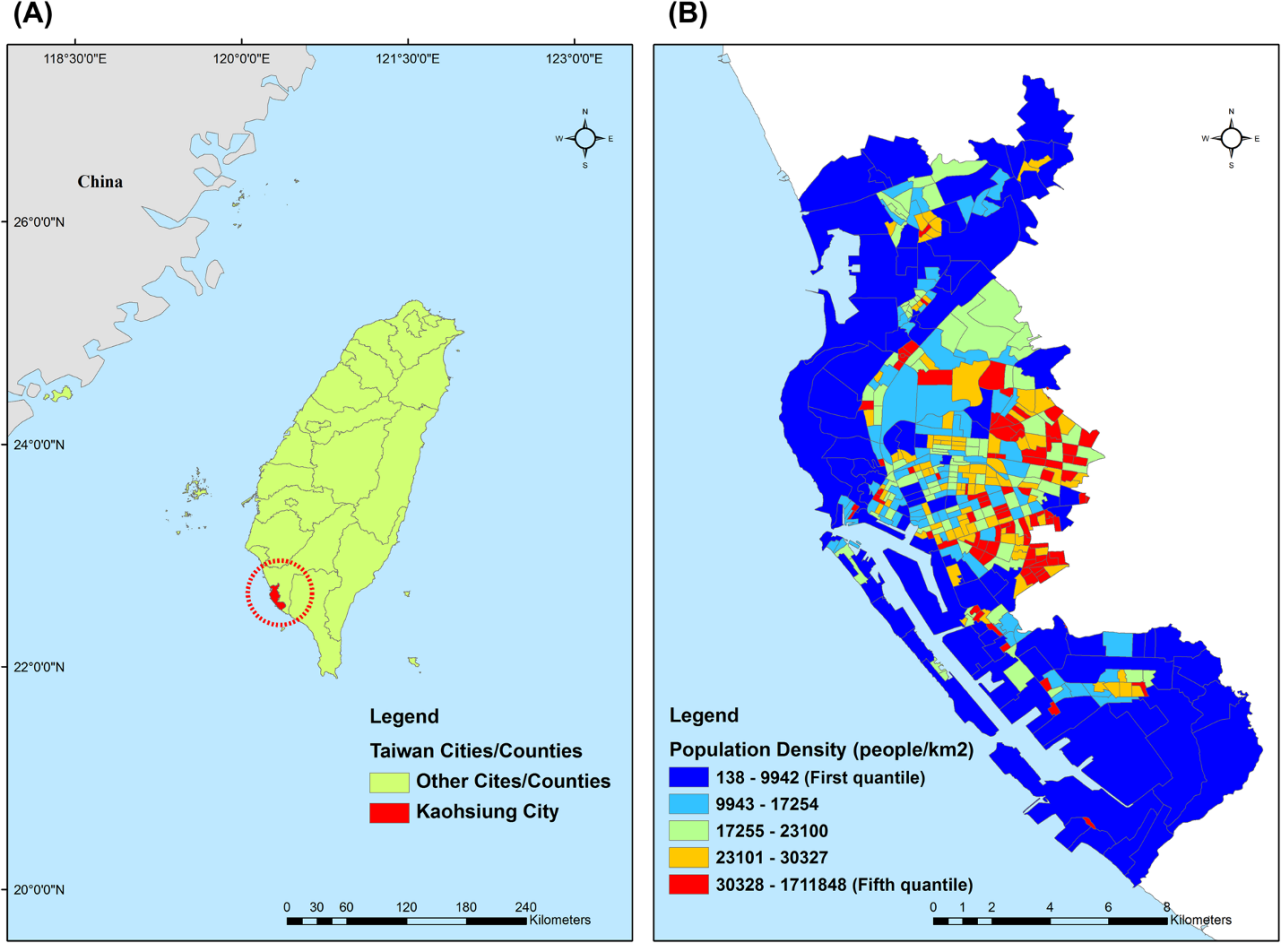
**The location of study area and the spatial distribution of population density. (A)** Kaohsiung City (filled with red color) is located in southern Taiwan. **(B)** The spatial distribution of the population density in Kaohsiung City. The classification of the symbology is based on the five quantiles of the population density.

The confirmed dengue cases were collected from the National Notifiable Diseases Surveillance System owned by the Taiwan Centers for Disease Control (CDC). The studied period is 2009 to 2012 and the studied area is the old section of Kaohsiung City (Figure 1-A). There are in total 2,997 confirmed dengue cases included in this study. The spatial unit of the surveillance is the village, with a mean size 0.36 km^2^ and a mean population density of 30,314 people/km^2^ (Figure 1-B). The population density was obtained by dividing the population in 2011 by the area size of each village, and the data were downloaded from the Taiwan socio-economic database maintained by the Ministry of the Interior (http://segis.moi.gov.tw/). In addition, we collected daily weather data, including precipitation and average temperature, from Taiwan’s Central Weather Bureau. There was only one weather station within the studied area.

### Ethics

This study was approved by the institutional review board (IRB) of Academia Sinica (IRB#: AS-IRB01-13060). The databases we used were all stripped of identifying information and thus informed consent was not needed.

### Statistical analysis

We denoted the daily reported dengue cases at the i^th^ urban village by *y*_*it*_ for the t^th^ day. Using the daily reported cases at the *n* villages in the city for *m* consecutive days before the current day, say *d* - 1, we constructed a logistic regression model for predicting the probabilities Pr(*Y*_*id*_ > 0) and determining a threshold to classify the villages as having cases or not on the d^th^ day. In addition to limited covariates such as population density and weather variables, we tried to construct various predictors using the reported cases in the affected villages. Basically, we considered three categories of autoregressive factors of cases reported in the four periods, which are the previous day, 2–7 days, 8–14 days and 15–30 days before the current day among the *n* villages. We decided these time intervals based on three criteria. First, we considered the extrinsic and intrinsic incubation period of dengue infection. The mean intrinsic incubation period (IIP) has been determined to be 5.9 days (95% CI: 3–10 days), with an extrinsic incubation period (EIP) at 25°C of 5 days to 33 days [18]. In general, 75% of infected patients have been found to develop symptoms by 7.1 days (95% CI: 6.7–7.6) [19]. That’s why we used 7 days for the first cut-points, and considered the EIP for the other 14 days. Second, we considered both the maximum lifetime of the *Aedes aegypti*, 22 days and IIP [20]. Thus, we decided on 30 days as our maximum observation window. Third, from a model selection perspective, we have also tried modeling with different combinations of time intervals, and found the current interval had good performance and is easy to remember in practical usage.

The first category of four predictors included average numbers of cases reported for the four periods at the same village. In notation, the four predictors for the i^th^ village and the t^th^ day were written as


The values of (*a*_*q*_, *b*_*q*_) are (1, 1), (2, 7), (8, 14) and (15, 30) for the four predictors. The second category included four similar predictors defined instead using the cases reported at the neighboring villages. Those were represented as


Where *N*_*i*_ is the set of villages which have common borders with the i^th^ village. These two categories of predictors were considered to reflect possible infection paths at the local level. To explore possible infection paths from other villages due to commuting or other regular movement activities, we first calculated average numbers of times the other villages reported cases during each of the four time periods after one village reported cases. These average numbers of times will be assigned as weights to the indicator variables of reporting cases of the villages for defining four predictors of the third category. Specifically, using the indicator function *I*(⋅), we defined four *n* × *n* matrix *W*_*q*_ of weights which elements are


for *j* ≠ *i*, *j* ∉ *N*_*i*_, and ; 0 otherwise. To have stable weights, the interval [*a*, *b*] has to be long, say covering data of three years, from 2009 to 2011. A large value of *w*_*qji*_ indicated that the i^th^ village had a high chance of having cases during [*a*_*q*_, *b*_*q*_] days after cases being reported in the j^th^ village. We then calculated the indicator variables , representing whether the j^th^ village reported cases during [*a*_*q*_, *b*_*q*_] days before the t^th^ day. The four predictors in the third category were defined as , which aggregates possible infection paths from the other villages to the i^th^ village. Due to the sparsity of the affected villages, we also defined two predictors of villages reporting no confirmed cases in the previous 30 days, denoted by , and in the neighboring villages, denoted by . We denoted population density of the village as *P*_*i*_ and defined four common weather variables for the villages. The first two indicator variables were given by *X*_1*t*_ = 1 (*X*_2*t*_ = 1) if daily precipitation between 5 mm and 50 mm (larger than 50 mm) occurred at least one day during the previous 21 days; 0 otherwise. The next two indicators were defined by *X*_3*t*_ = 1 (*X*_4*t*_ = 1) if daily average temperature higher than 29°C (lower than 20°C) occurred at least one day during the previous 7 days; 0 otherwise. The logistic regression model fitted to the *n* × *m* observations each day is then given by:


### Prediction

On day *d*, the logistic regression model was fitted to the dengue cases *y*_*it*_ for *i* = 1, 2, …, *n* and *t* = *d* - 1, …, *d* - *m*. We have considered four weather covariates in the regression model. If all the *m* values of a weather covariate are zero, the covariate is excluded from the model. We applied the glm function in the R package to estimate the model coefficients and calculated the *m* × *n* estimates of the probabilities Pr(*Y*_*it*_ > 0). We denoted *c*_*p*_ as the p^th^ percentile of those estimates of Pr(*Y*_*it*_ > 0) with *y*_*it*_ = 0. The value *c*_*p*_ will be used as a threshold for classifying predicted probabilities of the villages on the d^th^ day as having cases or not. The predicted probabilities can be obtained simply from the logistic regression model with the estimated coefficients and new predictors *A*_*qid*_, *B*_*qid*_, *C*_*qid*_, *Z*_*jid*_ and *X*_*jd*_. If the predicted probability at a village is larger than *c*_*p*_, we conclude that cases will occur at this village and none otherwise.

## Results

**Figure 2:**
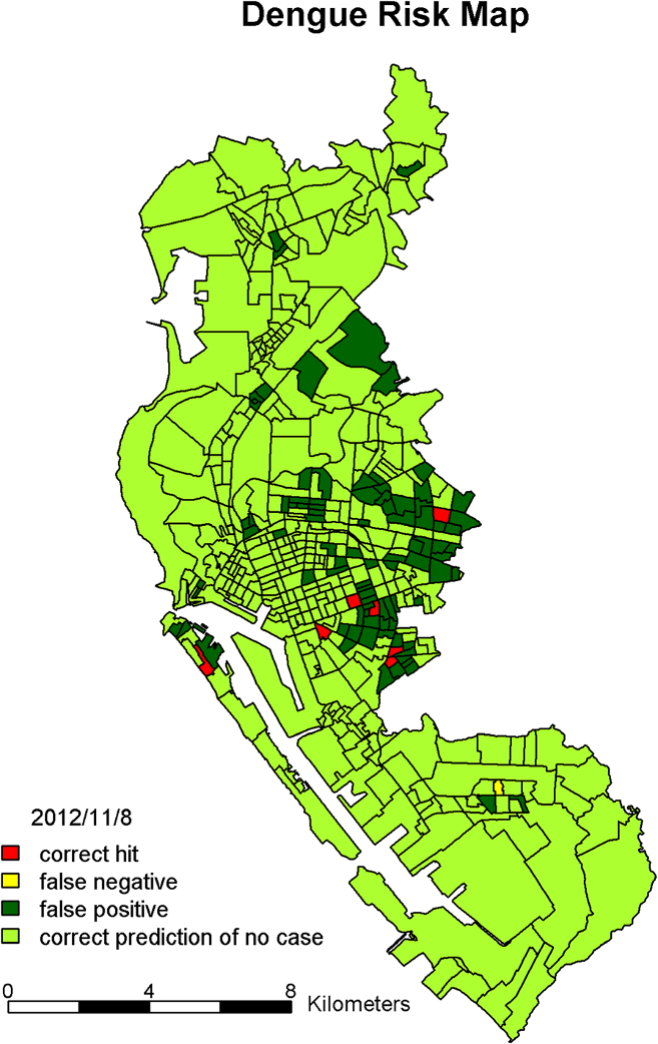
**The prediction results of dengue risk map of urban villages of Kaohsiung City.** The colors indicate prediction results on November 8, 2012: red is a correct hit; yellow is a false negative; dark green is a false positive; light green is a correct prediction of no case.

**Figure 3:**
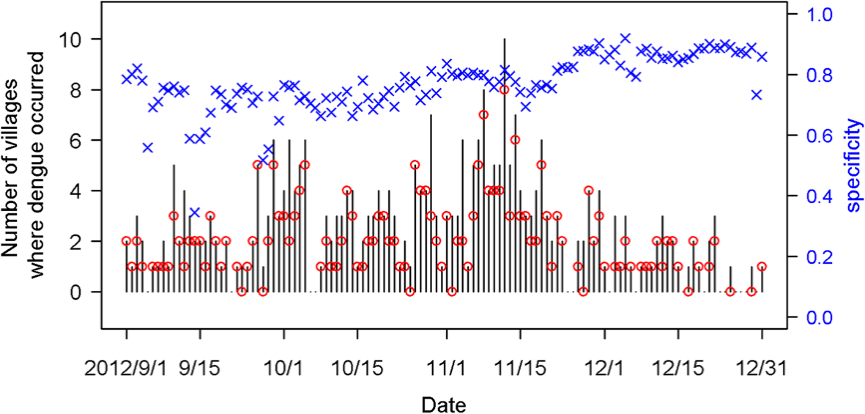
**Summary of predictions on the 457 villages for each day during September 1 to December 31, 2012.** Black line segments indicate observed number of villages reporting cases; red circles are numbers of affected villages correctly predicted; blue cross marks are percentages of dengue-free villages correctly predicted.

To examine the effectiveness of the predictors and assess the performance in prediction of the proposed method, we used the daily confirmed cases in 457 urban villages of Kaohsiung City from the years 2009 to 2012. We used three years’ data from 2009 to 2011 to compute the *W*_*q*_ matrix which is used as an indicator for exploring possible infection paths among different villages. That is, the first three years, 2009 – 2011, were used as our training data. Every year, the dengue epidemic begins to increase in severity after the rainy and typhoon seasons in July and August. Thus, we chose the period from September to December 2012 to validate our model’s performance. The evaluations were implemented repeatedly with consecutive *m* = 30 days of data before each day *d* from September 1 to December 31, 2012, which is the dengue epidemic season in Kaohsiung City. As an example, we show the prediction results of November 8, 2012 on the map with four colors (Figure 2). There were eight villages reporting dengue cases that day. The model gave seven correct hits (red) and one false negative (yellow). Although the model also issued a false positive on about 20% of the villages (dark green), half of them were close to dengue-affected villages. We summarize the prediction results from the 122 models during the four months in Figure 3. The daily numbers of villages with confirmed cases during the four months, ranging from none to 10 villages, are presented as in vertical segments. The numbers of dengue-affected villages correctly predicted for each day are marked with a red circle in Figure 3. In terms of the percentage of correct prediction, called the sensitivity, the median is 83% (Table 1-A). The percentage of dengue-free villages correctly predicted, called specificity (light green), is also shown in Figure 2. The median of these 122 specificities (i.e. 1 minus false positive rate) is 77% (Table 1-B). The results shown above were based on the cut-point *c*_80_. If *c*_75_ was adopted for classifying villages instead, the median sensitivity rose to 93% and median specificity went down to 72%.

**Table 1:** **Summary of** sensitivities **and** false positive rates **under different threshold cut-points from the 122 predictive models during September 1 to December 31, 2012**

(A) Sensitivities							
Summary statistics	Threshold cut-points						
	60%	70%	75%	80%	85%	90%	95%
Minimum	0	0	0	0	0	0	0
1st Quantile	0.75	0.667	0.6	0.6	0.5	0.333	0.333
Median	1	1	0.929	**0.833**	0.708	0.667	0.5
Mean	0.851	0.788	0.766	0.746	0.671	0.615	0.525
3rd Quantile	1	1	1	1	1	1	0.8
Maximum	1	1	1	1	1	1	1
(B) False positive rates							
**Summary statistics**	**Threshold cut-points**						
	**60%**	**70%**	**75%**	**80%**	**85%**	**90%**	**95%**
Minimum	0.143	0.101	0.086	0.082	0.062	0.022	0.009
1st Quantile	0.337	0.249	0.208	0.154	0.113	0.081	0.035
Median	0.42	0.321	0.28	**0.229**	0.173	0.117	0.06
Mean	0.43	0.331	0.282	0.228	0.175	0.118	0.058
3rd Quantile	0.497	0.395	0.335	0.273	0.213	0.142	0.072
Maximum	0.925	0.835	0.752	0.655	0.543	0.4	0.233

**Figure 4:**
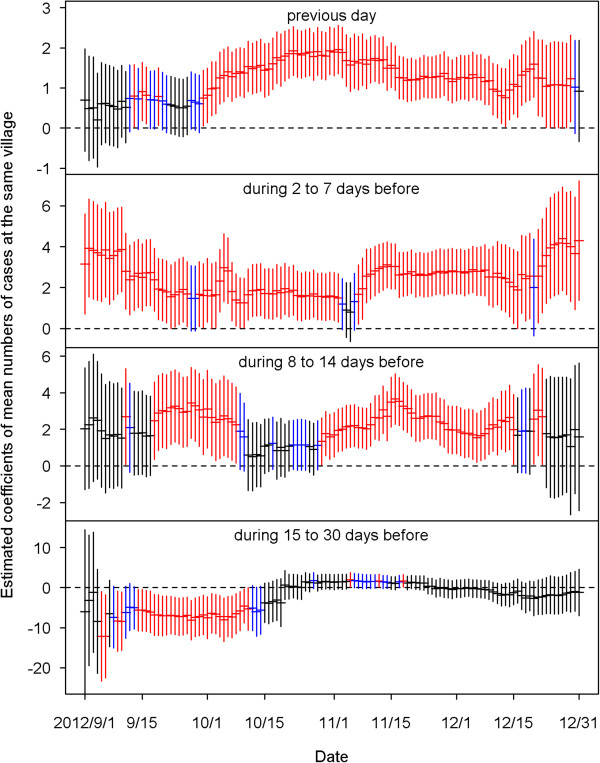
**The estimated coefficients with two standard errors of the predictors of mean numbers of cases at the same village on the previous day (top), during 2 to 7 days before, during 8 to 14 days before, and during 15 to 30 days before (bottom).** Black color indicates the estimation is not statistically significant (p≧0.1). Blue color indicates the estimation is marginally significant (0.05≦p < 0.1). Red color indicates the estimation is statistically significant (p < 0.05).

**Figure 5:**
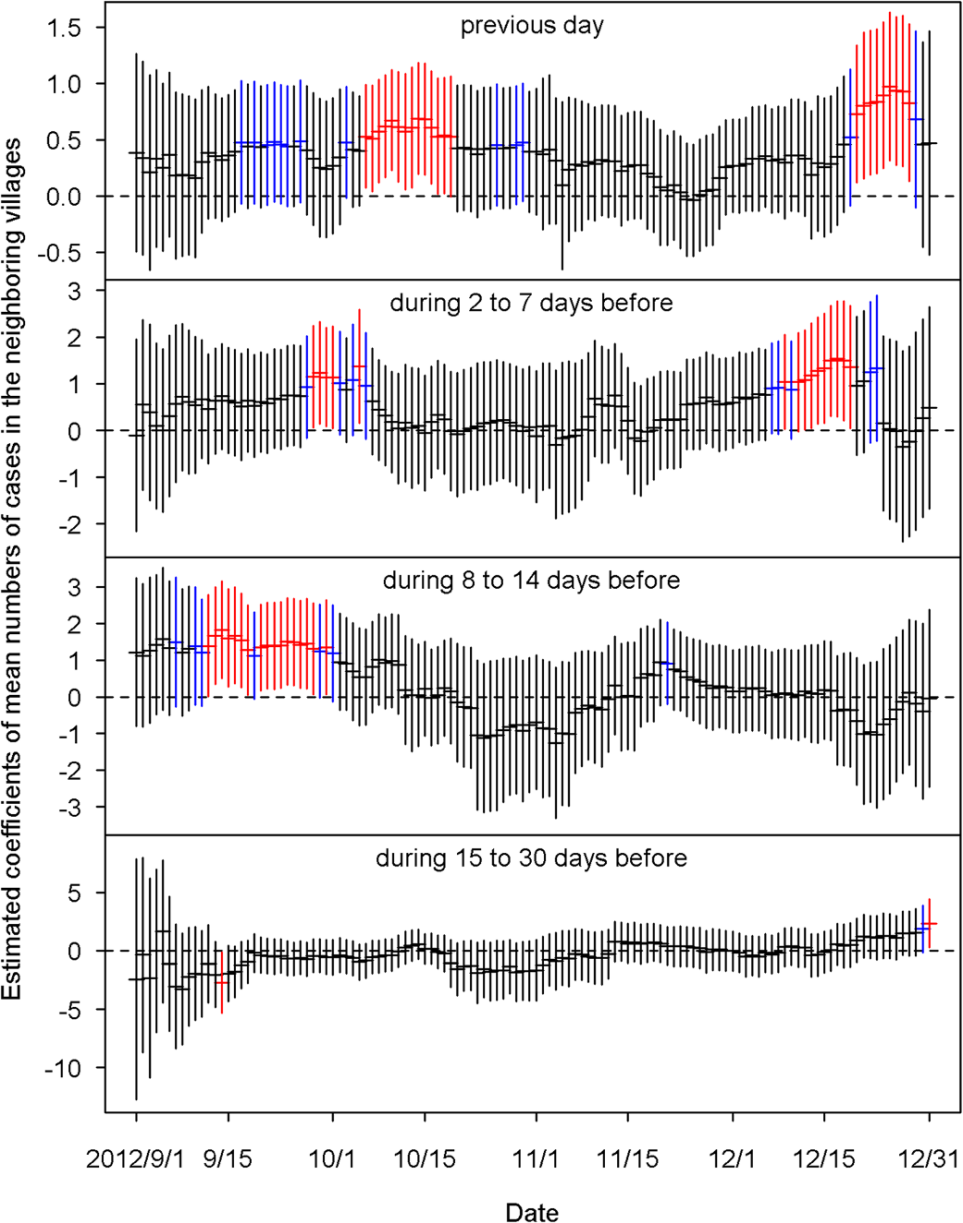
**The estimated coefficients with two standard errors of the predictors of mean numbers of cases in the neighboring villages on the previous day (top), during 2 to 7 days before, during 8 to 14 days before, and during 15 to 30 days before (bottom).** Black color indicates the estimation is not statistically significant (p≧0.1). Blue color indicates the estimation is marginally significant (0.05≦p < 0.1). Red color indicates the estimation is statistically significant (p < 0.05).

**Figure 6:**
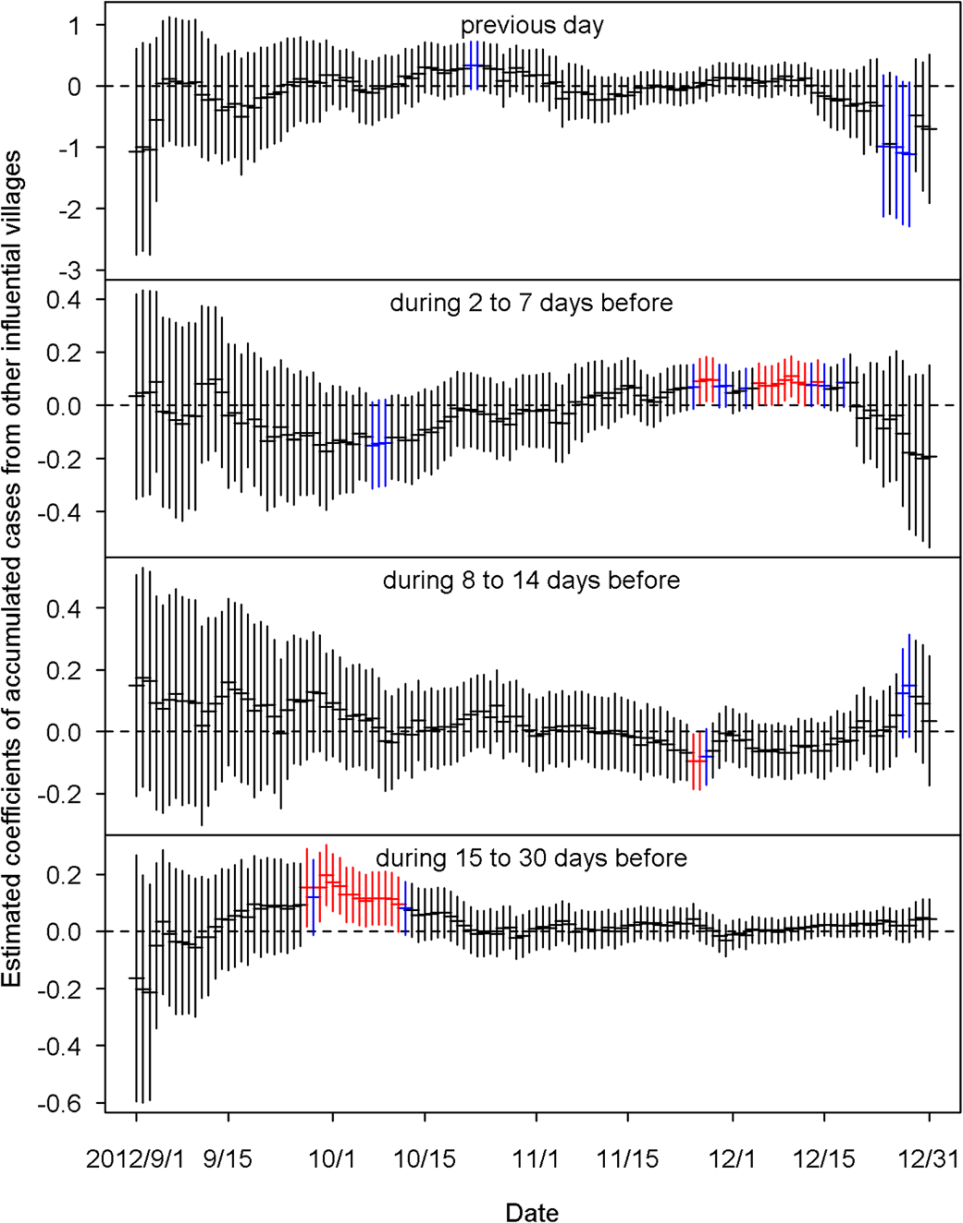
**The estimated coefficients with two standard errors of the predictors of accumulated cases from other influential villages on the previous day (top), during 2 to 7 days before, during 8 to 14 days before, and during 15 to 30 days before (bottom).** Black color indicates the estimation is not statistically significant (p≧0.1). Blue color indicates the estimation is marginally significant (0.05≦p < 0.1). Red color indicates the estimation is statistically significant (p < 0.05).

**Figure 7:**
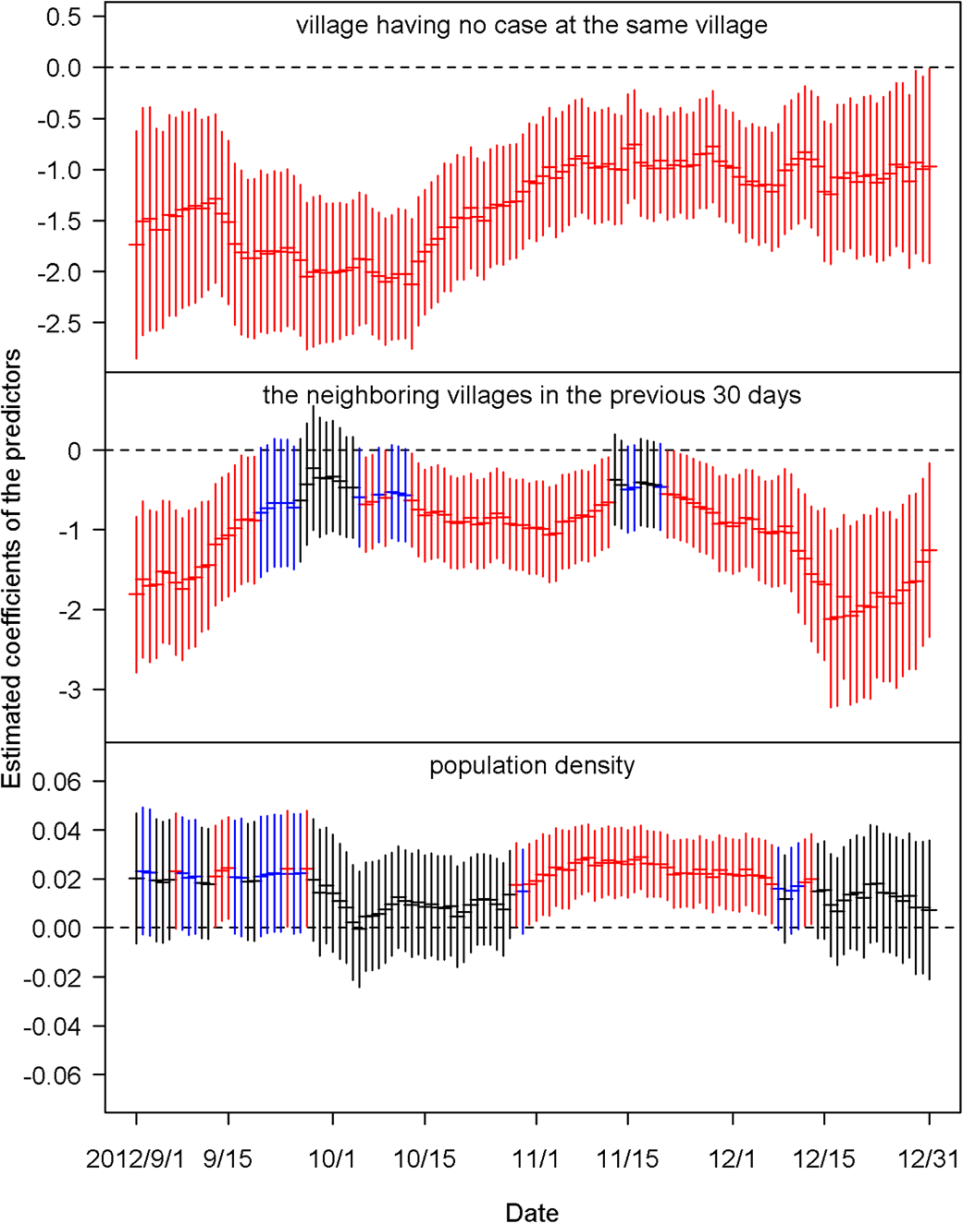
**The estimated coefficients with two standard errors of the three predictors for villages having no case at the same village (top), in the neighboring villages (middle) in the previous 30 days and population density (bottom).** Black color indicates the estimation is not statistically significant (p≧0.1). Blue color indicates the estimation is marginally significant (0.05≦p < 0.1). Red color indicates the estimation is statistically significant (p < 0.05).

Figure 4 shows the estimated coefficients of predictors defined by the average dengue cases of each village in the four time periods before the current day. We found that most of the coefficients of the predictors of having cases at the same village in the previous 14 days were positive and significant for the 122 models from September 1 to December 31, 2012. This indicates that when a case occurred in a village, the village had high odds of getting more cases in the coming 14 days. The bottom plot of Figure 4 shows that the strong positive effects after two weeks disappeared, and significantly negative effects were revealed before mid-October.The estimated effects from the neighboring villages are summarized in Figure 5. The top plot indicates that cases that occurred today at a village would increase risks for its neighboring villages tomorrow, although most of the estimated coefficients in the 122 days were insignificant. The influence from the neighboring villages decreased as lag time increased. Figure 6 shows the estimated coefficients with two standard errors of the predictors of accumulated cases from other possibly influential villages in the four time periods before the current day. Although most of them were insignificant, significantly positive estimates found on consecutive days around early October and December may indicate that some villages were affected by other villages via certain routes during those periods. The top two plots of Figure 7 show significantly negative estimated coefficients for predictors of villages having no confirmed cases in the previous 30 days in the same village and in neighboring villages. The results seem reasonable for the sparsity of affected villages during the four months. The bottom plot of Figure 7 shows significantly positive estimated coefficients of population density in November. This may reflect a dengue epidemic among the populous villages during those days. Most of the coefficients for the four weather-related predictors were not significant in the 122 fitted models during the four months of September – December.

## Discussion

In this study, we propose a small area-based dengue prediction method with timeliness improved by the daily prediction, like a weather forecast. In addition to the labor-intensive [21] and costly [22] vector surveillance, this sensitive, comprehensive and near real-time disease surveillance is also crucial to enhancing the accuracy of the prediction and improving risk management of dengue epidemics and outbreaks. One previous study found that implementing a rapid measure for controlling dengue outbreaks in the early stage can significantly reduce the epidemic size [23]. Therefore, this early warning statistical method will give front-line public health workers an advantage in identifying high-risk areas for intensive surveillance and early intervention.

Good quality of dengue surveillance is the basic infrastructure for any prediction model. In Taiwan, dengue is a category 2 notifiable infectious disease. The physicians have the responsibility to report the suspected dengue cases to the local health department within 24 hours of clinical diagnosis [24]. Thus, the quality of surveillance was ensured by the Communicable Disease Prevention Act, and the efficiency was enhanced by the internet reporting system in recent years. According to a previous study, the case fatality rate of dengue hemorrhagic fever (DHF) ranged from 9.1% to 50% during 2002–2007 [24]. The ratio of symptomatic and asymptomatic cases is 1.78 for adult dengue in Taiwan, which means about two-thirds of the dengue-infected adults have clinical symptoms [24]. Even when patients had clinical symptoms, a few cases would still be missed by physicians in hospitals [25]. Continuous education for physicians will be needed to reduce the under-reporting of dengue cases.

In the real situation, the dengue infected cases will be either asymptomatic or might be under-reported by the clinicians. Thus, when the surveillance system detects one confirmed case, it may mean some infected cases also exist in the same village or other villages. The current policy will initiate an epidemiological investigation after receiving a report of suspected or confirmed dengue cases, and the investigation is required to be completed within 24 hours [26]. At the same time, the environmental cleaning and mosquitos’ surveillance also need to be completed within 48 hours. The effect of peridomestic space *spraying does not persist very long. The major tasks suggested by Taiwan’s CDC* are source reduction [26, 27]. In relation to our prediction model, we can help the decision maker in the local health department to decide where the first priority villages to do intensive source reduction and environmental cleaning are. With these approaches, instead of chemical spraying, economic loss can be minimized and the participation and acceptance rate in the village will be elevated.

Unlike other dengue surveillance models with larger spatial and temporal units [28, 29], a small area or village (mean size: 0.36 km^2^) was our major targeted and surveillance unit. Therefore, we quantify the influence of local villages using different time lags. We found that the local influence was very strong from the previous day to 14 days. This was quite consistent with the incubation period of dengue infection [18]. We also found that when the epidemic became more severe, the estimated coefficient of the mean number of cases at the local village also increased positively and significantly. This finding meant that the local clustering and transmission was really present [30, 31]. The neighboring and other influential villages were not at all significant. However, including these two factors into the model will enhance the sensitivity and incorporate the risk estimation when the other villages have dengue infection. It might somewhat capture the underlying human movements. On the other hand, the specificity was also very important in minimizing false positives. In this study, we considered the fact that some villages had no confirmed cases of dengue in the previous 30 days, helping the model to identify and adjust for those low risk areas.

The tradeoff between sensitivity and false positive rate is always a difficult issue for the model prediction. In this study, the key point for determining these two values was by the thresholds or cut-points. The presented median sensitivity and false positive rate were obtained based on the threshold corresponding to an expected 20% false alarm rate. The sensitivity we presented here was 83% and specificity was 77% (1-false positive rate). In other dengue prediction models [28, 32], the sensitivities were around 60% and the specificities were around 97%. If we choose the threshold corresponding to an expected 10% false alarm rate, the sensitivity will decline to 66.7% but our specificity can be elevated to near 90%. Most of the other models were applied to weekly data and large regions or cities. The challenge for the small area and daily data in this study might be even higher than the current methods.

Although climate conditions such as temperature, precipitation, humidity, and sea surface temperature were found to be highly correlated with the cases of dengue infection [33–35], the influence of meteorological conditions was not detected in this study. Our study area was confined to one city with only one weather station. Kaohsiung City is located in the tropical climate zone, so weather conditions do not change very much with time, and there were no weather variations in the small areas we studied. This might be the reason why the weather conditions could not significantly explain dengue epidemics in small areas.

A complete dengue transmission network requires vectors (mosquitoes) and human beings. However, data from the vector surveillance were not systematically collected, and might be misleading if the surveillance was implemented after cases of dengue were reported or after insecticide spraying. Previous studies also found that the vector surveillance data were not good enough for identifying any correlation with dengue epidemics [11]. Therefore, we did not include this information into our final model. The other important factor was population density. One previous study found that around 3,000-7,000 persons/km^2^ in Vietnam was the condition for the highest risk for dengue epidemics, and the risk did not increase with density beyond that [17]. Some studies found that dengue cases clustered in urban areas [36]. In our studied area, the population densities were higher than the mentioned one in Vietnam. During our model validation period, the population density only showed a significant influence during the peak of the epidemic in 2012. High population density might only facilitate the dengue transmission cycle between human beings and mosquitoes when the number of infected cases is large enough, and the trend will still need to be validated by more data in the future.

### Limitation

Although this proposed method can be easily applied to routine dengue surveillance, there are still many challenges limiting the prediction power of the model. The first one is not knowing where the actual infection of each dengue case took place. The mosquitoes themselves cannot fly long distances, but the infected human may travel longer distances within the city or even beyond, and subsequently spread the infection. In this study, we have tried to use three different levels of influence, namely the local, neighboring, and other influential villages to capture possible impact of human movement. In this approach, we borrowed information mainly from the dengue surveillance data. If the information from epidemiological investigation can be applied to the surveillance model, the dynamic pattern of human movement might increase its prediction power. The second limitation is the lack of completed vector surveillance data. Although the current data were not suitable for the prediction, systematic collection of vector surveillance will be needed and might be beneficial for risk prediction in the future.

## Conclusion

Timely risk prediction and early intervention in the local villages are important and urgently needed to control dengue epidemics. With simple logistic regression and a dynamic alert threshold, sensitivity of nearly 80% and a false positive rate of 20% can be achieved in daily dengue risk prediction. The local effect of the same village was the significant predictor of dengue outbreaks within 14 days after the latest dengue infection occurred. In addition, this small area-based risk prediction will be practical for targeted surveillance and intervention. It will serve as a useful and near real-time decision-making tool for front-line public health workers. The precision of the spatial and temporal units can be easily adjusted to different settings for different cities.
